# Association Between Eccentric and Isometric Shoulder Rotation Strength, Shoulder Range of Motion and Injury Incidence in the Shoulder in Adolescent Competitive Tennis Players: The SMASH Cohort Study

**DOI:** 10.1177/19417381251396149

**Published:** 2025-11-21

**Authors:** Fredrik Johansson, Mark Batt, Todd Ellenbecker, Eva Skillgate

**Affiliations:** † Department of Health Promoting Science, Musculoskeletal and Sports Injury Epidemiology Center, Tennis Research and Performance Group, Sophiahemmet University, Stockholm, Sweden; ‡ Unit for Intervention and Implementation Research in Worker Health, Institute of Environmental Medicine, Karolinska Institutet, Stockholm, Sweden; § Scandinavian College of Naprapathic Manual Medicine, Stockholm, Sweden; ‖ Nottingham University Hospitals NHS Trust, Nottingham, UK; ¶ ATP Tour & Banner Sports Medicine, Scottsdale, Arizona

**Keywords:** adolescent, injuries, shoulder, strength, tennis

## Abstract

**Background:**

For competitive adolescent athletes, injury avoidance is a challenge, and causes of injury are complex and multifactorial. Despite an incidence of 8.2 shoulder injuries per 1,000 hours of tennis played, few studies have investigated the association between shoulder strength, range of motion (ROM), and injury.

**Hypothesis:**

Eccentric and isometric shoulder muscle strength and/or shoulder ROM are associated with new shoulder complaints and/or injuries.

**Study Design:**

Cohort study.

**Level of Evidence:**

Level 3.

**Methods:**

At baseline 301 adolescent competitive tennis players aged 13 to 19 years completed a questionnaire, were assessed with a shoulder protocol for strength and ROM and followed weekly (for shoulder complaint/injury) for 52 consecutive weeks. Outcomes were a first incidence of a tennis-related shoulder complaint or injury in the dominant arm, defined as a sum score of ≥20 or ≥40, respectively, on the Oslo Overuse Injury Questionnaire. Two cohorts were created for Cox regression analyses, adjusted for age, sex, and playing level: (1) shoulder complaints (n = 204), and (2) shoulder injuries (n = 252).

**Results:**

The most definitive adjusted associations were a hazard rate ratio (HRR) of 1.3 (95% CI 1.0-1.8) for a shoulder complaint in eccentric external rotation (eccER) strength, and a HRR for shoulder injuries of 1.4 (95% CI 1.0-1.9) in isometric internal rotation (IIR) strength in the 90-90 position, and 1.5 (95% CI 1.1-2.0) in eccER normalized to body mass.

**Conclusion:**

Higher values of eccER shoulder strength, IIR strength at the 90-90 position, and eccER shoulder strength normalized to body mass, were associated with shoulder complaints/injuries in adolescent competitive tennis players.

**Clinical Relevance:**

Incorporating a training program that takes volume and intensity into account in the daily oncourt sessions, to build resilience through a well-planned, long-term training and competition plan to minimize shoulder injury risk may be of importance.

For competitive adolescent athletes, injury avoidance is a huge challenge, and the causes for injury are complex and multifactorial.^57^ Sport specialization has become more common in many sports, including tennis, which likely increases the risk of injury.^28^ The incidence and prevalence of injuries in the tennis population based on cross-sectional data, prospective studies, and systematic reviews presents a range between 3 and 20 injuries per 1000 hours played.^14,19,20,26,34,40,46-48^ Moreover, overuse injuries with a weekly prevalence of 12.1% constitute the most common form of injury in adolescent tennis players.^47^ More specifically, with an incidence of 8.2 injuries per 1000 hours played, the dominant shoulder represents 15.9% of the overuse injuries in the competitive adolescent tennis player.^19,47^

Publications on risk factors in overhead sports such as handball and tennis are emerging.^1-3,6,7,13,27,39,42,51,52,54,55^ Intrinsic risk factors (RF), such as a history of shoulder pain with or without shoulder injury, decreased rotational range of motion (ROM) in internal rotation (IR), increased ROM in external rotation (ER) and total range of motion (TROM), ER muscle weakness and muscular shoulder ER/IR imbalances,^6^ as well as extrinsic factors such as field position, match or training load,^30,41,43^ and training frequency in overhead athletes are associated with an increased shoulder injury risk.^6,30,41,43^

Previous studies point to the onset of physical adaptations in the adolescent tennis player,^10^ which are almost exclusively in the dominant arm (DA), defined as the arm exposed for the overhead service motion. Specific clinical adaptations in strength in the DA measured with a hand-held dynamometer (HHD), such as a decrease in ER strength, and imbalances such as low intermuscular ratios between ER/IR strength have been presented.^11,17,22,51^ Furthermore, rotational ROM in both IR and TROM are decreased, while ER ROM is increased in the DA.^11,17,22,51^ In addition to clinical assessments of adaptations, radiologic changes have also been reported including growth plate alterations,^31^ bony adaptations,^24^ and tendon changes.^32^

Even though these early adaptations occur and may affect the adolescent tennis player in different ways, not all these DA adaptations are well understood, and at present there is no clear association with injury.^31,32^

The shoulder is placed under high load in tennis but especially during the overhead service motion.^16^ In the late cocking phase, the shoulder is externally rotated to 170° and then moves rapidly into IR after ball impact, reaching 2420 deg/s and 1370 deg/s for male and female players, respectively.^16,18^ For competitive adolescent players, who are mastering the technique and the effective use of the kinetic chain, the speed of the serve can reach 145 kph to 160 kph, generating high loads in the underdeveloped shoulder.^35^ In addition, shoulder IR and ER strength seem to correlate with peak serve speed, underlining the importance of a balanced shoulder complex for high-speed serving.^25^ Furthermore, since a match and/or training session performed daily can include up to 100 to 120 serves,^43^ resilience against high training volumes needs to be considered in view of shoulder complaints and/or injuries.

Although there are publications documenting prevalence and incidence of injuries, and identification of apparent risk factors, very few studies in tennis are based on larger cohorts investigating injury causation over a longer time with regards to the shoulder.^20,26,30,40,43,44,47^

Therefore, the primary aim of the current study was to investigate whether shoulder eccentric and isometric muscle strength and/or ROM, are associated with shoulder complaints and/or injuries in adolescent competitive high-level female and male tennis players.

It was hypothesized that shoulder strength measures of IR, ER, abduction (ABD), eccentric strength, and ROM may identify adolescent tennis players at risk of shoulder injuries.

## Methods

### Data Collection

This study was based on data from the SMASH cohort study (Shoulder Management and Assessment Serving High Performance), collected in Sweden in February 2018 to March 2019, in which 301 adolescent competitive tennis players, 176 boys and 125 girls, mean age 14.6 (±2.0) and 14.4 (±2.0) years respectively, volunteered to participate. The players comprised regional (n = 251) and national (n = 50) level players from all 7 regions across Sweden. National players were defined as the top 8 players in the Swedish tennis association high-performance program.

An informed consent form was read and signed by the players, if <15 years of age, the players’ legal guardian read and signed the consent form.

**Figure 1.**
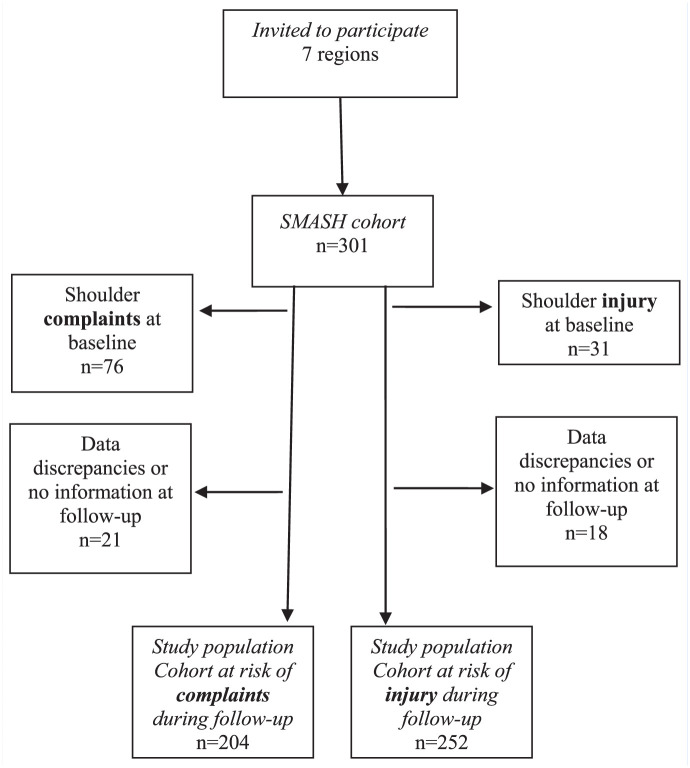
Flowchart describing inclusion process. SMASH, shoulder management and assessment serving high performance.

Inclusion criteria were (1) a minimum average of 8 hours of total training volume per week, and (2) competitive level of at least regional level in Sweden. In the full SMASH cohort (n = 301) there was no exclusion initially, but, for risk analyses, only players without a shoulder injury (OSTRC-O cutoff score 40 of 100) or complaint (OSTRC-O cutoff score 20 of 100) in the 3 months before the baseline testing were included as described (Figure 1).

A baseline questionnaire was completed, and players were followed prospectively for 52 consecutive weeks regarding the outcome via an App with weekly questionnaires and a reminder 24 hours later if a response had not been received. The average weekly response rate of the follow-up questionnaires was 85%, with 51% reporting complete data, 68% reporting at 90% of the follow-ups, 79% reporting 75% of the follow-ups, and 85% reporting at least 50% of the follow-ups.

### Exposure—Testing Procedure at Baseline

The exposure of interest in this study was isometric shoulder strength in IR and ER at shoulder level with 0° of ABD, isometric shoulder strength of IR and ER at 90° of ABD, isometric shoulder ABD in the scapular plane, eccentric external rotation (eccER) shoulder strength in an abducted position from 90° of ER to 0° of ER, and ROM for IR and ER ROM were performed in the coronal plane of ABD with scapular stabilization.^7,9,33^

#### Testing Procedure

The testing procedure at baseline was performed during a single visit by 1 of 3 teams consisting of 3 assessors per team. All teams were trained before testing by an experienced clinician and user of the HHD.

Initially, a comprehensive questionnaire was filled in by the players, including questions such as training and competition load, strength and conditioning training, and years of playing tennis.

Second, before the warm-up and shoulder testing, the players body mass was assessed on a digital scale, shoes and heavy clothing were removed. Thereafter, a supervised and standardized 10-minute warm-up program was performed, consisting of several multiplanar shoulder movements as well as flexibility exercises, preparing the player for the strength and ROM shoulder testing. Both the shoulder protocol and warm-up program has been published and described in detail recently,^29^ and will therefore only be described in overview.

### Protocol

#### Glenohumeral Muscle Strength

For all strength measurements, the MicroFET HHD was used (MicroFet 2, Hoggan Health Industries Inc, Biometrics). The order of tests was randomized between sides to minimize learning effects and fatigue.

The shoulder protocol consisted of 6 different isometric strength tests and were assessed at 0° and 90° of ABD for both IR and ER strength. ABD was tested in the scapular plane, and eccER was tested at 90° of ABD.^29^ All 6 tests were performed on the DA and non-DA (NDA) independently.

For eccentric testing with the HHD, the participant was in a seated position holding the elbow in 90° of flexion, and the shoulder in 90° of ABD and 90° external rotation (90-90) with light support from the clinician’s hand. The HHD was positioned 2 cm proximal to the ulnar styloid process and placed on the dorsal side of the forearm.^33^ The participant performed a maximal external rotation force while the examiner pushed the arm from the 90-90 position to 90° of ABD in neutral rotation (90-0) with a speed of 30 deg/s.^33^

The first examiner measured isometric strength (N) with the HHD, and the second examiner registered the test value to the test-protocol. Each test was repeated 2 times with a pause in between trials of 20 seconds.^9,29^

The shoulder protocol using the HHD has shown good-to-excellent intra- and interrater reliability ranging between 0.87 and 0.91 for ROM and 0.92 and 0.95 for strength measurements.^9,33^

#### Glenohumeral IR and ER ROM

The smartphone inclinometer app, GetMyRom (Version 1.0.3; Interactive Medical Productions) was used to assess shoulder internal and external passive ROMs. Both the dominant and nondominant shoulder were measured, using methods described previously in the literature.^36^ These procedures have previously shown good test-retest reliability and excellent intra- and interrater reliability.^9^

### Outcomes Measures

The outcomes were a first incidence of a tennis related shoulder complaint in the DA, defined as a sum score of ≥20,^31^ and a first incidence of a tennis-related shoulder injury in the DA, defined as a sum score of ≥40 on the Oslo Overuse Injury Questionnaire (OSTRC) at some point during follow-up.^8^

### Study Population

For the incidence analyses in this study, 2 cohorts were created; one for the incidence of shoulder complaints over time (n = 204), where players with shoulder complaints at baseline were excluded, and one for the incidence of shoulder injuries over time (n = 252), where players with shoulder complaints and injuries at baseline were excluded. Players who had not answered any weekly and follow-up questionnaire were excluded from both cohorts (n = 21 in the complaint cohort; n = 18 in the injury cohort). This was performed to be able to study a population at risk of shoulder injury.

### Potential Confounders

We measured potential confounders at baseline, and age, sex, and playing level were considered confounders in the association between exposures and outcomes.

### Statistical Analyses

#### Descriptive Statistics

Characteristics regarding the study sample (Table 1) are presented as means and standard deviations or proportions and numbers.

**Table 1.** Descriptive characteristics of study population at baseline

Baseline characteristics	Risk cohort for shoulder complaints (Cohort 1) (n = 204)	Risk cohort for shoulder injuries (Cohort 2) (n = 252)	All (n = 301)
Age, years	14.3 (1.9)	14.4 (2.0)	14.5 (2.0)
Sex, % boys (n)	57 (116)	57 (155)	58 (176)
Height, cm	168.4 (10.6)	169.3 (11.0)	169.8 (11.2)
Weight, kg	57.1 (12.0)	57.8 (12.5)	58.3 (12.7)
BMI, mean	19.9 (2.5)	19.9 (2.5)	20.0 (2.5)
Passion for sport (AIMS)^1^	28.8 (3.5)	28.9 (3.7)	29.0 (3.7)
Quality of sleep^2^	8.2 (1.5)	8.0 (1.6)	8.0 (1.7)
Number of hours of sleep per night	8.2 (1.4)	8.2 (1.5)	8.1 (1.5)
General health^2^	8.5 (1.5)	8.4 (1.7)	8.3 (1.7)
Matches in 2017, n	63.4 (32.5)	63.1 (33.7)	64.5 (35.1)
Tennis training per week in 2017, hours	9.3 (3.8)	9.4 (3.8)	9.5 (3.8)
Fitness training per week in 2017, hours	3.7 (2.4)	3.8 (2.5)	3.8 (2.5)
Normal racket tension, kg	23.4 (1.4)	23.4 (1.3)	23.4 (1.3)
A responsible tennis coach, % yes (n)	72 (146)	67 (182)	66 (200)
A responsible fitness coach, % yes (n)	66 (134)	64 (172)	62 (186)
Regularly performing rotation exercises for shoulder, % yes (n)	53 (108)	56 (152)	57 (172)

Data presented as mean (SD). AIMS, athletic identity measurement scale; BMI, body mass index.

#### Inferential Statistics

Injury incidence was calculated as the numbers of complaints/injuries divided by 1000 hours of matches/training exposure.

All exposures were standardized with a mean of 0 and a SD of 1. The hazard rate (HR) estimates correspond to the increase in hazard when the exposures are increased by 1 SD.

We built multivariable Cox proportional hazard models, one for each exposure, to compute HR ratios (HRRs) and 95% CI for the association between the exposures and the event of the first complaint/injury. Participants’ time at risk corresponded to the number of hours of tennis training and match play from baseline until the first complaint or injury event. This information was given in the weekly follow-up questionnaires. Players who reported a shoulder complaint/injury or chose to leave the study were censored in the Cox proportional hazard models.

Our modelling strategy included 2 steps. First, we performed a crude analysis, 1 model for each exposure. Second, we adjusted for confounders. The proportional hazard assumption (eg, that the multiplicative effect of the hazard function is constant over time) was held for all models (Schoenfeld residuals) and was tested for each model. No power analysis was performed.

### Ethical Statement

The study was in accordance with the declaration of Helsinki and preapproved by the Regional Ethical Review Board, Stockholm, Sweden (2012/1731/2 and 2018/2510).

## Results

Descriptive characteristics of the study population are presented in Table 1. The mean age was 14 years, and 57% were boys.

**Figure 2.**
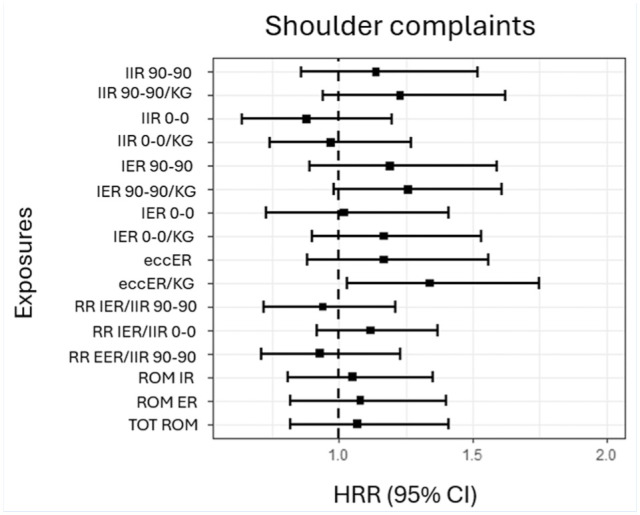
HRRs for shoulder complaints for strength and ROM measures, adjusted for age (continuous), sex, and playing level (national or regional). eccER (kg) normalized to body weight. Test position of abduction for the glenohumeral joint (90-90 or 0-0) noted for IIR and IER. ABD, abduction; IIR, isometric IR; IER isometric ER; eccER eccentric ER; RR, rate ratio; ROM, range of motion; TOT, total ABD; HRR, hazard rate ratio.

The HRRs for shoulder complaints are presented in Figure 2. The most definitive associations were an adjusted HRR of 1.3 (95% CI, 1.0-1.8) for a shoulder complaint for each increase of 1 SD in eccER strength, and an adjusted HRR of 1.3 (95% CI, 1.0-1.6) for a shoulder complaint for each increase of 1 SD in isometric ER (IER) strength in the 90-90 position, normalized to body mass.

**Figure 3.**
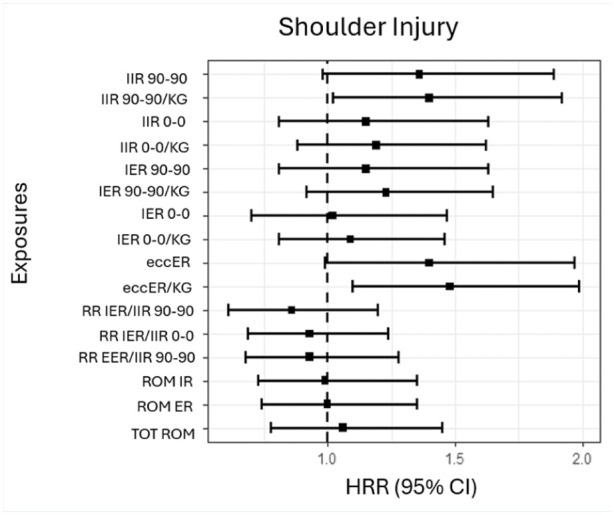
The HRR for shoulder injuries for strength and ROM measures, adjusted for age (continuous), sex, and playing level (national or regional). eccER (kg) normalized to body weight. Test position of abduction for the glenohumeral joint (90-90 or 0-0) noted for IIR and IER. ABD, abduction; IIR, isometric IR; IER isometric ER; eccER eccentric ER; RR, rate ratio; ROM, range of motion; TOT, total ABD; HRR, hazard rate ratio.

The HRRs for shoulder injuries are presented in Figure 3. The most definitive associations were an adjusted HRR of 1.4 (95% CI, 1.0-1.9) for a shoulder injury for each increases of 1 SD in isometric internal rotation (IIR) strength in the 90-90 position, an adjusted HRR of 1.4 (95% CI, 1.0-1.9) for a shoulder injury for each increases of 1 SD in isometric internal rotation (IIR) strength in the 90-90 position normalized to body mass, an adjusted HRR of 1.4 (95% CI, 1.0-2.0) for a shoulder injury for each increase of 1 SD in eccER strength, and an adjusted HRR of 1.5 (95% CI, 1.1-2.0) for a shoulder injury for each increase of 1 SD in eccER strength, normalized to body mass.

## Discussion

### Main Results

The principal finding of this cohort study was that increased IIR strength at the 90-90 position and eccER shoulder strength normalized to body mass are associated with a higher incidence of shoulder complaints/injuries in adolescent competitive tennis players. We did not find any association between either IR ROM or TROM in the shoulder, indicating that, in terms of injury risk, this is not of importance for shoulder complaints and injuries in this cohort.

### Isometric Internal Rotation Strength as a Risk Factor

In the current study, the results reveal that IIR shoulder strength assessed in the 90-90 position is a risk factor, which stands in contrast to results reported in previous systematic reviews.^34,46,55^ During the adolescent years, the growth and maturation of the young athlete leads to changes in the neuromuscular system, for example, the architecture of the muscle-tendon and muscle activation.^49,56^ Furthermore, the tendon in growing athletes has a slower adaptation and response to mechanical load in comparison with the muscle characterized by a greater rate of tissue renewal; therefore, the tendon is potentially more at risk for complaints/injuries then the muscle itself.^37^ Moreover, IIR shoulder strength and neuromuscular fitness have been shown to be correlated to increases in strength and serve speed.^4,5,21,45^ Based on these previous findings, adolescent competitive tennis players will most likely use IR shoulder strength as a primary component to perform high-speed serving, and since the tendon at this young age most likely is underdeveloped in relation to the muscle they may experience complaints/injuries in the shoulder area, as has been published in baseball.^38,53^ From a clinical perspective, this means that IR shoulder strength needs to be adressed, but an understanding of the tendons response in relation to the muscle becomes important to balance the training load so that the tendon has time to adapt to the load produced in high-speed serving by the muscles performing shoulder IR.

### Eccentric External Rotation Strength as a Risk Factor

In this specific cohort of adolescent tennis players, players that were stronger in eccER shoulder strength are more susceptible to injury, although it seems counterintuitive at first, it makes more sense when we explore the larger context. First, as stated earlier, IR shoulder strength is correlated to higher serve speed in tennis despite the serve being a complex biomechanical stroke consisting of a series of segmental rotations occurring though the kinetic chain. Therefore, players that are strong in shoulder IR will at the same time develop eccER shoulder strength since the overhead service motion in tennis and the forehand stroke both demand high activity of the external rotators in the deceleration movement of the shoulder.^50^

Second, tennis in general constitutes high training volumes in hours per week,^47^ including high numbers of repetitions in groundstrokes as well as serving. Subsequently, higher racket speeds and a high volume in external training load, in combination leads to a greater stress in the primary tissues such as the muscles, tendons, and skeletal tissues and would impact injury risk. However, since these explosive movements are repeated thousands of times during a competitive season and most likely cause substantial fatigue in the eccER shoulder movement,^15^ this may create complaints/injuries in the shoulder.

This scenario in comparison with a weaker shoulder/player that cannot produce high forces and racquet head velocities and therefore will not to the same extent expose the shoulder to high loads, may help to explain the counterintuitive finding in this study regarding increased eccER shoulder strength and elevated injury risk.

Clinically, this means that, although an eccER shoulder assessment at a specific point in time, such as performed in the HHD testing, shows high numbers, it does not necessarily mean that the shoulder over time can withstand the thousands of repetitions in tennis and be resilient against the external load created. Therefore, to measure the peak force of eccER using a HHD as performed in our study, although previously shown to be reliable and valid in comparison with the isokinetic gold standard assessment,^33^ may not be the optimal and stand-alone assessment for tennis. Instead, a fatigue test may be a better option for future studies trying to understand the importance of repetitive eccER strength in adolescent overhead athletes such as tennis players.^12^

### Range of Motion

Despite the fact there are studies reporting on decreased ROM as a risk factor for shoulder injury, our cohort study could not confirm those findings. This may be for different reasons, as our cohort is relatively young and therefore adaptations in ROM are not yet to be considered a risk factor. During the adolescent years, the unilateral and repetitive forces on the dominant side may impact the shoulder complex, resulting in a decreased IR ROM.^22^ In a recent systematic review and meta-analysis including 2522 adolescent athletes, an association between glenohumeral internal rotation deficit and shoulder or elbow injury could not be established.^23^ As these results are conflicting to some extent, it means that, from a clinical perspective, ROM cannot be assessed and analyzed as a single parameter but needs to be evaluated in a larger context.

### Methodological Considerations

In research, trying to explore factors associated with an incident of an injury, the longitudinal study design, where exposure measurements (strength and ROM) are taken before the outcome (an incidence of a shoulder complaint/injury), is a strength. Also important for studying incidence is that cohorts used for these analyses are created in which only players without shoulder complaints/injuries at baseline are included. Both these conditions were true for the present study. The fact that the response rate was high on the weekly follow-up questionnaires (overall weekly average of 85%) indicates a low risk of selection bias. Nevertheless, if there is an association between loss to follow-up and shoulder muscle strength or shoulder ROM at baseline, which we find unlikely, there is a risk of an under- or overestimation of the associations investigated. We find it more likely that this loss to follow-up was not related to the exposure status and therefore did not constitute a threat to the study validity. The widely used and validated OSTRC questionnaire helped lower the risk of misclassification of outcomes.^8^ The adjustment for potential confounders in the analysis of the association between exposures (muscle strength and ROM) and outcome (shoulder complaint/injury) is also a strength, even though the risk of unmeasured and residual confounding cannot be ruled out. There might be factors associated with muscle strength/ROM and the risk of complaint/injury that differ between exposed and unexposed players that can partly explain the associations we found. Another potential limitation is the possible change in exposure status over the 52-week follow-up. Even though players and coaches were totally unaware of the test scores at baseline, if they were inferior or superior to other players in strength scores, we cannot rule out that the players have altered their strength during the period from baseline to the first event of a substantial shoulder injury. Players classified as stronger at baseline might have become weaker at follow-up, and players classified as weaker at baseline might have become stronger at follow-up. The most probable is that this potential change in exposure status is random, that is, not associated with whether they become injured later or not. This nondifferential misclassification of exposure, may have resulted in a dilution of the true association between the exposure and the outcome, meaning that the association we have found between a weaker strength and a later incident of shoulder injury are, most probably, underestimated—the true HRR should be higher. One possible explanation regarding our results could be weaker players becoming stronger during the follow-up period of the study. For this to be plausible, the change in strength would have to be quite large such that these weaker players became stronger on average than the players classified as the strongest at baseline. However, we find this explanation unlikely. The size of this study’s cohort is relatively large, including most elite players at this level in Sweden at the time of data collection. Despite this large cohort, there are limitations in the precision of the data, with wide CIs and associations.

## Conclusion

Higher IIR strength at the 90-90 position, and eccER shoulder strength normalized to body mass, are associated with a higher incidence of shoulder complaints/injuries in adolescent competitive tennis players. Shoulder ROM measures were not of importance for the incidence of shoulder complaints and injuries in this cohort.

### Practical Implications

Based on our findings, the stronger the player, the higher the risk for complaints/injury in the shoulder. Therefore, it may be important to structure the daily on-court practice taking volume and intensity into account, especially sessions that include a significant number of overhead repetitions such as serving. Furthermore, off-court strength training should aim to create resilience against repetitive work, not only focusing on peak force/strength, but also incorporating a shoulder endurance strength protocol as well. Finally, to further minimize the risk of injury, an overall well-planned training and competition plan is crucial to avoiding spikes in load to ensure long-term development of the adolescent tennis player.
